# Intraoperative Appearance of Endosalpingiosis: A Single-Center Experience of Laparoscopic Findings and Systematic Review of Literature

**DOI:** 10.3390/jcm11237006

**Published:** 2022-11-27

**Authors:** Laurin Burla, Dimitrios Rafail Kalaitzopoulos, Anna Mrozek, Markus Eberhard, Nicolas Samartzis

**Affiliations:** Department of Gynecology and Obstetrics, Hospital of Schaffhausen, 8208 Schaffhausen, Switzerland

**Keywords:** endosalpingiosis, mullerianosis, endosalpingiosis morphology, endosalpingiosis distribution, laparoscopy, minimal-access surgery

## Abstract

Background: Endosalpingiosis is assumed to be the second most common benign peritoneal pathology after endometriosis in women. Although recent studies indicate a significant association with gynecologic malignancies, many underlying principles remain unclear. This work aimed to systematically describe the intraoperative appearance of endosalpingiosis. Methods: Data and intraoperative videos of patients with histologically verified endosalpingiosis were retrospectively reviewed. The main outcome measures were macroscopic phenotype and anatomical distribution. Additionally, a systematic review searching PubMed (Medline) and Embase was conducted. Results: In the study population (n = 77, mean age 40.2 years (SD 16.4)), the mean size of lesions was 3.6 mm and the main visual pattern was vesicular (62%). The most frequent localization was the sacrouterine ligaments (24.7%). In the systematic review population (n = 1174 (210 included studies overall), mean age 45.7 years (SD 14.4)), there were 99 patients in 90 different studies with adequate data to assess the appearance of the lesions. The mean size of the lesions was 48.5 mm, mainly with a cystic visual pattern (49.5%). The majority of the lesions affected the ovaries (23.2%), fallopian tubes (20.4%), or lymph nodes (18.5%). Comparing this study to the literature population, the main differences concerned the size (*p* < 0.001) and main visual patterns (*p* < 0.001) of lesions. Conclusions: The usual intraoperative findings of endosalpingiosis appeared less impressive than described in the literature. In our study population, lesions of a few millimeters in size with a vesicular appearance were mostly seen, most frequently in the sacrouterine ligament area. Intraoperative recognition by the gynecologic surgeon and histologic diagnosis should play an important role in further understanding this entity, scientifically and clinically.

## 1. Introduction

Endosalpingiosis is the ectopic presence of a fallopian tube-type glandular epithelium and has a prevalence of ~7% in premenopausal women [1,2]. Described by Sampson in 1930 as “post-salpingectomy endometriosis”, endosalpingiosis has long been perceived as an insignificant incidental finding, and thus the relevance of this condition remains largely unknown [3].

Endosalpingiosis is the second most common peritoneal disease in women following endometriosis, the most common representative of the condition known as mullerianosis [4]. These two entities occur concurrently in about 30–40% of cases [1,5]. A comparative study showed that endosalpingiosis does not have a chronic inflammatory nature and does not cause infertility or chronic pelvic pain compared with endometriosis [1]. The etiology remains unexplained for both to date; analogous theories have been discussed such as retrograde menstruation, metaplasia of the coelomic epithelium, embryonic misplacement, and hematogenous or lymphomatous dissemination [4,6]. Apart from exceptions where extensive lesions are revealed on imaging, endosalpingiosis is usually detected intraoperatively.

However, recent work indicates that endosalpingiosis is associated with gynecological tumors including uterine and ovarian neoplasia; among the latter, especially serous borderline, clear cell, invasive mucinous tumors, and endometrioid cancer subtypes [5,7], sharing similar molecular pathomechanisms [8].

Systematic descriptions of macroscopic appearance are sparse, making it difficult to recognize the lesions and distinguish them from endometriosis or other findings intraoperatively. Similar reference works as for endometriosis hardly exist [9].

Here, we systematically investigated the intraoperative macroscopic phenotype and anatomical distribution of endosalpingiosis based on an own patient population and a systematic literature review.

## 2. Materials and Methods

### 2.1. Own Population

The study was designed in compliance with the STROBE checklist [10]. Data and intraoperative videos of patients with endosalpingiosis undergoing a laparoscopy between 2007 and 2020 in the Department of Obstetrics and Gynecology of Cantonal Hospital Schaffhausen were examined. Exclusion criteria were a lack of histologically verified endosalpingiosis and missing or insufficient intraoperative video material. Every included video was reviewed by two reviewers independently (AM, NS). In cases of disagreement between the two reviewers, a third reviewer (LB) was invited to participate, and the consensus was reached by discussion.

### 2.2. Sample Size Calculation

The minimum required sample size was calculated based on the study by Hesseling et al. [2], presumably the most comprehensive description of intraoperative findings in endosalpingiosis to date, versus a review of the current literature data. According to these data, lesions of 1 to 10 mm in diameter seem most frequent in clinical routine. This is in line with our experience and in contrast to the literature, where mostly larger findings of 4–5 cm have been reported. Regarding the anatomical distribution, in the study by Hesseling et al., the majority of lesions were in the pouch of Douglas with 69%, whereas only 7% were seen there in the literature review. In consideration of these findings, assuming a statistical power of 80% (*p* = 0.05), at least 18 participants in each group were required to describe the macroscopic phenotype, or eight for the anatomic distribution.

### 2.3. Systematic Review Population

This systematic review was conducted according to the PRISMA Guidelines [11]. The study protocol was registered in PROSPERO (CRD42022303171). The search for eligible studies was conducted in two databases (PubMed, Medline, and Embase) using a combination of the following MeSH terms as an electronic search algorithm: Endosalpingiosis OR Mullerianosis OR Endometriosis after salpingectomy. Reference lists of relevant articles and associated reviews were manually searched to identify papers not captured in the electronic search. Original studies (cohort studies, case-control studies, case reports) concerning humans in any language were considered for inclusion. Studies were included if their focus was on endosalpingiosis and if they contained information about the macroscopic appearance and/or the anatomical distribution. Exclusion criteria were an insufficiently precise description of the appearance (in words or pictures), missing anatomical information, or a lack of histological confirmation.

If the same cases were included in more than one publication (e.g., abstract and full-text manuscript), only the publication with the most detailed information was considered. Abstracts providing information about the macroscopic presentation and anatomical distribution of endosalpingiosis were considered eligible if no full-text manuscript was available.

The main search was conducted independently by three investigators (LB, DRK, NS) for the relevant literature published until 31 December 2021. Discrepancies were resolved by consensus. In addition to information on the general characteristics of the studies (authors, year of publication, journal, design, number of patients), the following parameters were recorded in standardized Excel spreadsheets.

### 2.4. Parameters

In both the own population and the systematic review population, the clinicopathological characteristics of the patients (e.g., age, parity, menopausal status, previous abdominal or gynecological surgery, indication for surgery, concurrent endometriosis and/or cancer) were recorded.

The primary endpoint was the macroscopic phenotype of endosalpingiosis lesions; the secondary endpoint was the anatomical localization.

**Figure 1:**
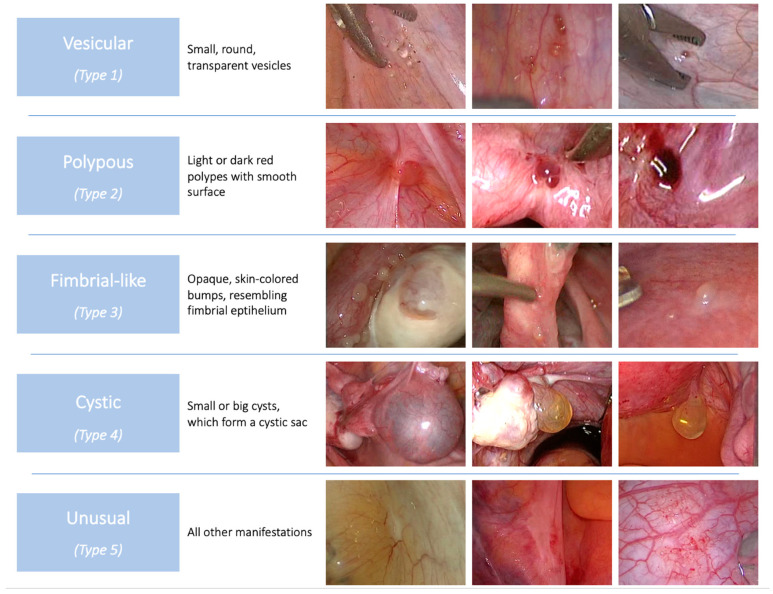
Based on the morphologic criteria, lesions were classified into five visual patterns.

The appearance of the lesions from patients was described in terms of the shape, color, height, surface area, consistency, associated calcifications/adhesions/fibrosis, and histological presence of endometriosis in the same lesions. On this basis, lesions were allocated into five main visual patterns (types 1–5: vesicular, polypous, fimbrial-like, cystic, and unusual). This classification has been described previously by our group (Figure 1) [12].

### 2.5. Statistical Analysis

Statistical analyses were performed with IBM SPSS Statistics 27 (Endicott, NY, USA). For the categorical data, the Chi-square test was used; for continuous data, the Mann–Whitney U test was used. *p*-values < 0.05 were considered statistically significant.

### 2.6. Quality Assessment Systematic Review

Quality assessments for the included studies were conducted independently by three reviewers (LB, DRK, NS). Quality assessment for the observational cohort studies was performed using the Newcastle–Ottawa Scale and, for case reports, the JBI critical appraisal checklist for case reports [13,14].

### 2.7. Patient and Public Involvement

Apart from the retrospectively recorded, anonymized laparoscopic images and clinicopathological data of patients of the own population, there was no patient or public involvement in this study. Patient consent was obtained for the anonymous re-use of the data and intraoperative images. Approval for research was obtained from the local ethics committee (2020-02718). There are no conflicts of interest to declare.

## 3. Results

### 3.1. Own Population

#### 3.1.1. Demographic Data—Age, Parity, Menopause, Reasons for Surgery

In our study group, we found 77 patients with histologically verified endosalpingiosis. The mean age was 40.2 years (SD 16.4), and the mean BMI was 24.1 kg/m^2^ (SD 5.7). Most (75.3%, n = 58) patients where premenopausal. Most (59.7% n = 46) were nulligravida and (70.1%, n = 54) nullipara. Of the 23 women who gave birth, 30.4% (n = 7) had at least one cesarean section. Close to half (46.7% n = 36) did not have any previous abdominal or vaginal surgery; 29.9% (n = 23) were smokers; 59.7% (n = 46) did not take any kind of hormonal treatment at the time of surgery; 13% (n = 10) were on combined oral contraceptives, 15.6% (n = 12) on the progestogen-only pill or had a levonorgestrel intrauterine device, 3.9% (n = 3) had GnRH-analogues, 1.3% (n = 1) ulipristal acetate, 1.3% (n = 1) bromocriptine, and 2.6% (n = 2) had a hormonal replacement treatment.

Endometriosis was simultaneously present in 53.2% (n = 41) of all cases. According to the American Society for Reproductive Medicine (ASRM) endometriosis classification, 46.3% (n = 19) were at stage I, 14.6% (n = 6) at stage II, 9.8% (n = 4) at stage III, and 29.3% (n = 12) at stage IV. Gynecological malignancies were associated in 28.6% (n = 22); among them, there were seven cases of endometrial cancer, one case of uterine carcinosarcoma, eight cases of borderline ovarian tumors, five cases of epithelial ovarian cancer, and one case of yolk sac tumor of the ovary (Table 1).

Reasons for surgery were in most cases pelvic pain (29.9%, n = 23), surgery for gynecologic malignancies (27.3%, n = 21), infertility (20.8%, n = 16), and suspicious pelvic mass (15.6%, n = 12). All indications are shown in Table 2. Of the 77 patients, 6.5% (n = 5) underwent colorectal surgery (one rectal segmental resection, four shaving of the rectal muscularis).

#### 3.1.2. Phenotype

Most (64.9%, n = 50) of the cases could be adequately visualized. Five cases were excluded because laparotomy was performed without video documentation, seven cases because no video was archived, and three cases were due to poor video quality. In seven patients, endosalpingiosis could not be distinguished on the peritoneum or from other adjacent lesions (i.e., endometriosis). Endosalpingiosis was not visible due to its sole location in the lymph nodes (three cases) or omentum (two cases).

**Figure 2:**
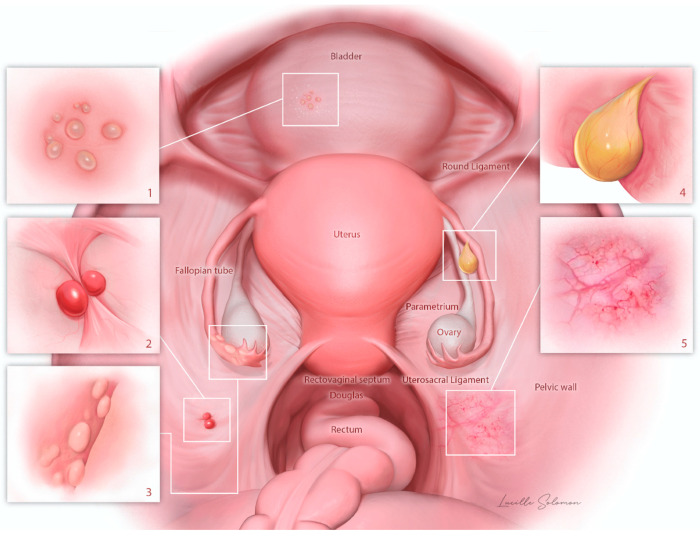
Schematic representation of the different patterns of endosalpingiosis in the female pelvis as seen by the laparoscopist (patterns 1–5 according to Figure 1).

The mean single-lesion size was 3.6 mm (range 1–40 mm, SD 5.7 mm). The main colors were transparent (48%, n = 24) and white (22%, n = 11). Most lesions had a regular shape (64%, n = 32), were flat (70%, n = 35), had a smooth surface (84%, n = 42), and had a soft or liquid consistency (88%, n = 44). Calcifications were present in 24% (n = 12), and adhesion in 32% (n = 16). The main visual group was vesicular type (62%, n = 31), followed by fimbrial-like (12%, n = 6), cystic (10%, n = 5), and polypous (6%, n = 3) (Table 3). In three cases, there was a second lesion of endosalpingiosis. Among them, two were vesicular and one of type 5 (unusual). Figure 2 provides a schematic intraoperative view of the findings.

#### 3.1.3. Anatomical Distribution

Adequate information on anatomic distribution was available in all 77 cases. Twenty-six percent (n = 20) were multicentric, meaning that they were found in more than one localization. Most (89.6%, n = 69) were located in the pelvis, 14.3% (n = 11) in the remaining abdominal cavity, and 3.9% (n = 3) in the lymph nodes. The most frequent localization was the sacrouterine ligaments (24.7%, n = 19), followed by the peritoneum of Douglas (20.8%, n = 16), and of the bladder (19.5%, n = 15) (Table 4).

### 3.2. Systematic Review

**Figure 3:**
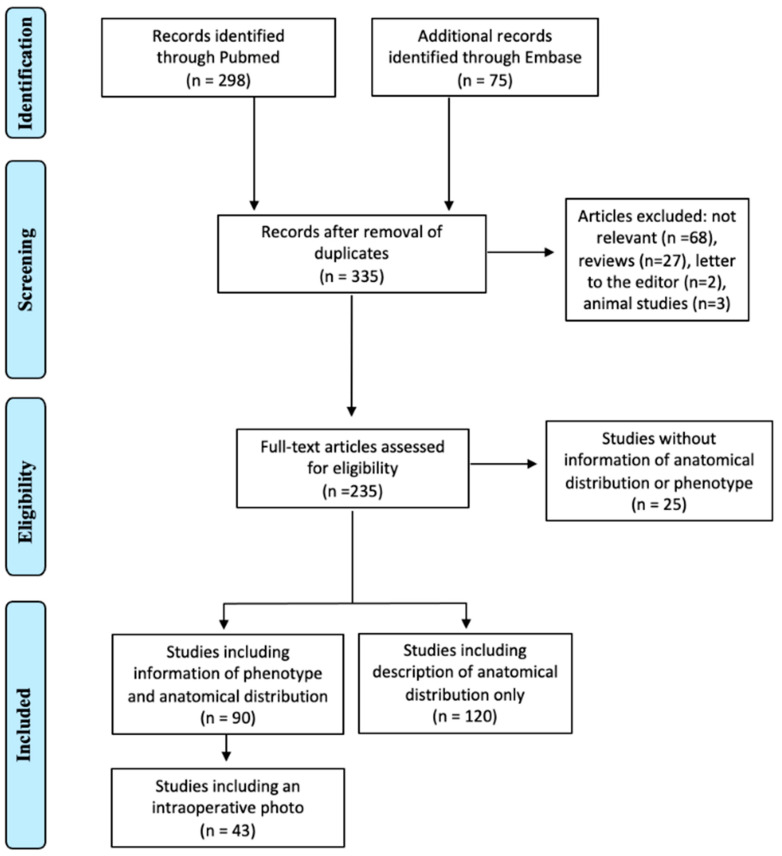
PRISMA flow diagram of the study selection in the systematic review population.

Two hundred and ten publications were included, with a total of 1174 patients. Among them, 77.1% (n = 162) were case reports or case series with less than five cases, and 22.9% (n = 48) of publications were original human research. Less than half (42.8%, n = 90) of the articles had information about the visual aspect of endosalpingiosis and anatomical distribution, and 20.5% (n = 43) of the studies included a picture of the macroscopic appearance [2,15,16,17,18,19,20,21,22,23,24,25,26,27,28,29,30,31,32,33,34,35,36,37,38,39,40,41,42,43,44,45,46,47,48,49,50,51,52,53,54,55,56,57,58,59,60,61,62,63,64,65,66,67,68,69,70,71,72,73,74,75,76,77,78,79,80,81,82,83,84,85,86,87,88,89,90,91,92,93,94,95,96,97,98,99,100,101,102,103] (Figure 3). Most (81.4%, n = 171) included a histological picture, 8.1% (n = 17) an ultrasound image, 11.0% (n = 23) a CT-scan, and 11.4% (n = 24) an MRI image. More than half (57.1%, n = 120) of the studies included only information on the anatomical distribution and no depiction of the phenotype [1,8,104,105,106,107,108,109,110,111,112,113,114,115,116,117,118,119,120,121,122,123,124,125,126,127,128,129,130,131,132,133,134,135,136,137,138,139,140,141,142,143,144,145,146,147,148,149,150,151,152,153,154,155,156,157,158,159,160,161,162,163,164,165,166,167,168,169,170,171,172,173,174,175,176,177,178,179,180,181,182,183,184,185,186,187,188,189,190,191,192,193,194,195,196,197,198,199,200,201,202,203,204,205,206,207,208,209,210,211,212,213,214,215,216,217,218,219,220,221]. All of the studies with information on the phenotype also indicated the anatomical distribution.

#### 3.2.1. Demographic Data—Age, Parity, Menopause, Reasons for Surgery

The mean age of the patients was 45.7 years (SD 14.4). We found information about menopausal status in a total of 88 patients. Most (65.9%, n = 58) were premenopausal. Data about parity was available for 50 patients. Twenty-two percent (n = 11) were nulliparous, 20% (n = 10) primiparous, and 58% (n = 29) had more than one child.

Of the 1174 included patients, only 9.4% (n = 110) had a malignancy. Among them, 19.1% (n = 21) was ovarian neoplasm, 30.9% (n = 34) was uterine cancer, 17.3% (n = 19) was breast neoplasm, 6.4% (n = 7) was cervical neoplasm, 2.7% (n = 3) was intestinal neoplasm, and 15.5% (n = 17) was others. There were no reported vaginal or vulvar cancer in the included studies. Ovarian neoplasm included borderline tumors and ovarian cancer. Breast neoplasm included ductal carcinoma in situ and breast cancer. There was no information about the exact entity in the remaining 8.1% (n = 9) (Table 1).

Data on the indication for the surgery was available in 295 patients. These were mainly: 28.8% (n = 85) suspicious pelvic mass, 21.4% (n = 63) acute or chronic pelvic pain, 15.6% (n = 46) gynecologic neoplasm, and 7.1% (n = 21) fertility diagnostic. All indications are shown in Table 2. Of the 295 patients where indication for surgery and procedure were known, 3.7% (n = 11) received colorectal surgery (seven rectosigmoid resections, one right hemicolectomy, one ileocecal resection, one other colonic segmental resection, one small bowel segmental resection).

#### 3.2.2. Phenotype

In 99 patients in 90 different studies, enough data were present to evaluate the macroscopic appearance. The mean size was 48.5 mm (range 2–250 mm). Most of the lesions were irregular in shape (63.6%, n = 63), transparent (31.3%, n = 31), or dark in color (25.3%, n = 25), cystic (76.8%, n = 76) with a smooth surface (88.9%, n = 88), and liquid consistency (67.7%, n = 67). The main visual group was the cystic type (49.5%, n = 49), followed by unusual (30.3%, n = 30), polypous (11.1%, n = 11), vesicular (8.1%, n = 8), and finally fimbrial-like (1%, n = 1) (Table 3). In four cases, there was a second type of phenotype. Three of them additionally had a type 4 (cystic), and one a type 5 (unusual) lesion.

#### 3.2.3. Anatomical Distribution

In 210 publications with a total of 1174 patients, there was information about the lesion localization of the endosalpingiosis lesion. In 90.6% (n = 1064) of the cases, the lesion was localized only on one site, affecting not more than one organ (unilocular); 4.9% (n = 57) were multilocular; and 4.5% (n = 53) were diagnosed on abdominal washing cytology and could not be assigned to the above two groups. The most frequent localization was the ovaries (23.2%, n = 272), the fallopian tubes (20.4%, n = 239), and the lymph nodes (18.5%, n = 217). Table 4 shows all the different localizations of the lesion.

### 3.3. Comparison between Own and Systematic Review Population

When comparing the own with the literature population, there were significant differences in the macroscopic aspect between our collective and the reported cases in terms of size (*p* < 0.001), shape (*p* = 0.001), color (*p* = 0.005), height (*p* < 0.001), consistency (*p* = 0.007), adhesions (*p* = 0.002), and in the main visual groups (*p* < 0.001) (Table 3).

Furthermore, there were significant differences in the anatomical distribution. We found more lesions on the peritoneum of the bladder (*p* < 0.001), the parametrium (*p* < 0.001), the sacrouterine ligaments (*p* < 0.001), the pelvic sidewall (*p* < 0.001), the cavity of Douglas (*p* < 0.001), and the abdominal wall (*p* = 0.047). In contrast, the cases reported in the literature were more likely to be localized in the ovary (*p* < 0.001), fallopian tube (*p* < 0.001), and lymph nodes (*p* < 0.001) (Table 4). There was no significant difference in the percentage of colorectal procedures between the two populations (*p* = 0.287).

## 4. Discussion

This study shows the relevant differences between the own population, reflecting clinical practice at a gynecological reference center, and the systematic literature population.

In both populations, the main indications for surgery were pelvic pain, gynecological neoplasm, infertility, and pelvic mass. Significantly more frequent in the own population was fertility diagnostic and surgery for neoplasms; in the literature group, it was pelvic mass. That fertility work-up is a common indication for surgery in patients with findings of endosalpingiosis is consistent with Prentice et al. (27.6% (n = 16/58) vs. 27.1% (16/59) in premenopausal patients) [1]. That pelvic mass was more common as an indication in the literature population is most likely due to the large manifestations seen in preoperative imaging [73,81,213]. The indications seem heterogeneous, which strengthens the currently accepted thesis that endosalpingiosis is mostly an incidental finding and does not cause pain or infertility [1].

This study’s clinically most relevant finding lies in the macroscopically different lesions (Table 3). Based on nine phenotypic features described in the Materials and Methods, the lesions were subdivided into five visual patterns, which have been published elsewhere [12]: Type 1 lesions (vesicular) are mostly smaller than 5 mm, symmetric with a translucent clear or yellow liquid content; Type 2 (polypous) are around 5 mm to 10 mm in size, with a smooth surface and reddish color with the closest resemblance to endometriosis; Type 3 (fimbrial-like) looks like fimbrial mucosa with a smooth opaque surface and appears as grouped bumps, frequently on fallopian tubes; Type 4 (cystic) are usually bigger than 10 mm, forming a cystic sac and can be found as pedunculated structures attached to pelvic organs. Type 5 includes all other lesions. The average size of a single lesion in the study population was less than 4 mm (mostly vesicular (Type 1), 62%), whereas in the literature population, it was almost 5 cm (mostly cystic (Type 4), 49.5%) (Table 3, Figure 1). This difference most likely resulted from publication bias. Most (77.1%) of the articles containing information on the intraoperative aspect of endosalpingiosis are case reports, where impressive examples are interesting [66,68]. This underlines that knowledge on the part of the laparoscopist is important to even recognize this entity intraoperatively.

To date, the origin of endosalpingiosis is not clear. Similar hypotheses of development (retrograde menstruation, metaplasia of the coelomic epithelium, iatrogenic, metastatic, embryonic remnant) are proposed as for endometriosis. Reactive excessive tubal proliferation following salpingitis is another theory [4,222]. Additionally, the natural history of endosalpingiosis and the course of changes over the lifespan are completely unclear.

The term “florid” endosalpingiosis is frequently used in the literature, representing large cystic findings. A recognized definition is missing, so it is unclear whether the term “florid” correlates with biological behavior [51,68,74,78,87,200].

How and even whether to approach endosalpingiosis lesions surgically has not been determined. We used near-contact laparoscopy with high resolution. All macroscopically detected foci were removed by local peritoneal excision. Due to the unclear significance and for histological workup with differentiation from other entities, especially endometriosis, until now, we have deliberately decided to avoid ablative procedures.

Although the distinction from endometriosis can often only be made histologically, some features can help to differentiate these entities intraoperatively. According to our experience and published hypothesis, endosalpingiosis lesions seem to have a more symmetrical, clearly circumscribed shape, sometimes surrounded by fine adhesions (32%) and calcifications (24%). Endosalpingiosis seems rarely associated with inflammation (neoangiogenesis, fibrosis). Additionally, we did not encounter any distortion of the anatomy in our cases, as is common in deep endometriosis [12].

The calcifications look like grains of sand and are associated with psammoma bodies, which are dystrophic calcification following cellular degeneration [223]. These are in ovarian serous papillary neoplasms and non-neoplastic peritoneal diseases such as endosalpingiosis [224].

Another differential diagnosis to be considered is peritoneal mesothelioma. This presents from smaller peritoneal lesions of 2 to 20 mm to larger cystic findings. On immunohistological examination, these lesions can be distinguished from endosalpingiosis [160,225,226].

Concerning localization, 90% of lesions in our own population were located in the pelvis, most commonly on the sacrouterine ligaments, bladder, or the remaining cavity of Douglas. In the literature, more lesions were on the ovaries or fallopian tubes and as incidental findings of lymphadenectomies. Hesseling et al. found the most common lesions in the cavity of Douglas, followed by the cardinal ligaments [2].

In the literature population, the prevalence of malignancy in patients with endosalpingiosis was significantly lower than in the own population, which showed similar frequencies to the epidemiologic studies by Hermens et al. and Esselen et al. [5,7,227]. It is possible that large cystic forms are less frequently associated with malignancy. It is still unclear whether endosalpingiosis is an insignificant incidental finding or represents a relevant risk factor or even a precursor lesion [154]. As recently published research has shown, there is increasing evidence that most low-grade serous tumors in the ovary are related to endosalpingiosis [228].

The limitations of this study—concerning the own population—were the retrospective monocentric design, the limited study population, and that we could not guarantee that all manifestations were seen. To detect as many lesions as possible, we adopted the concept of near-contact laparoscopy [229]. Concerning the retrospective design and limited population, it can be said that endosalpingiosis is still usually an incidental finding, so there is no preoperative inclusion in an endosalpingiosis cohort. In addition, biopsy and optimal imaging are required for this type of study. Nevertheless, a sample size calculation was performed to have enough power for the research question. Regarding the systematic review, a relevant proportion of the literature consisted of case reports; there were hardly any similar works to compare.

This work raises questions that could be addressed in the future: Do different types of endosalpingiosis actually exist, and is this reflected at the histopathologic level? Is there a different neoplastic potential?

## 5. Conclusions

The endosalpingiosis lesions found in clinical practice are much less prominent than those described in the literature. These are often a few millimeters in size, vesicular in appearance, and located in the small pelvis. For further scientific and clinical understanding of endosalpingiosis including its association with malignancy and the resulting recommendations for clinical consequences in the future, detailed knowledge of endosalpingiosis among gynecologic surgeons as initial diagnosticians is essential.

## Figures and Tables

**Table 1:** Demographic data in the own and the systematic review population.

			Own Population	Systematic Review	*p*-Values
Age (years)			40.2 (SD 16.4)	45.7 (SD 14.4)	0.003
Menopausal status					0.232
	premenopausal		75.3% (58/77)	65.9% (58/88)	
	postmenopausal		24.7% (19/77))	34.1% (30/88)	
Parity					<0.001
	0		70.1% (54/77)	22% (11/50)	
	I		7.8% (6/77)	20% (10/50)	
	≥II		22.1% (17/77)	58% (29/50)	
Endometriosis			53.2% (41/77)	9% (106/1174)	<0.001
	rASRM	I/II	61% (25/41)	56.3% (9/16 *)	
		III/IV	39% (16/41)	43.8% (7/16 *)	
Neoplasm **			28.6% (22/77)	9.4% (110/1174)	<0.001
	cervical		1.3% (1/77)	6.4% (7/110)	
	uterine		10.4% (8/77)	30.9% (34/110)	
	ovarian		18.2% (14/77)	19.1% (21/110)	
	breast		2.6% (2/77)	17.3% (19/110)	
	intestinal		2.6% (2/77)	2.7% (3/110)	
	other		2.6% (2/77)	15.5% (17/110)	

SD—Standard deviation. * Only 16 cases in which the rASRM stage is indicated. ** Neoplasm also includes non-invasive entities such as cervical cancer-in situ, endometrial hyperplasia, and borderline ovarian tumors. rASRM = Revised Score of the American Society for Reproductive Medicine.

**Table 2:** Reasons for surgery in the own and the systematic review population.

	Own Population (n = 77)	Systematic Review (n = 295)	*p*-Values
Pelvic pain	29.9% (n = 23)	21.4% (n = 63)	0.115
Gynecologic neoplasm *	27.3% (n = 21)	15.6% (n = 46)	0.018
Fertility diagnostic	20.8% (n = 16)	7.1% (n = 21)	<0.001
Suspicious pelvic mass	15.6% (n = 12)	28.9% (n = 85)	0.019
Abnormal uterine bleeding	3.9% (n = 3)	5.1% (n = 15)	0.665
Bowel disorder	1.3% (n = 1)	1.4% (n = 4)	0.969
Risk reduction surgery	1.3% (n = 1)	1.0% (n = 3)	0.831
Breast cancer	0% (n = 0)	6.1% (n = 18)	0.026
Suspicion of urinary tract neoplasm	0% (n = 0)	5.1% (n = 15)	0.043
Urinary tract disorder	0% (n = 0)	3.7% (n = 11)	0.085
Inguinal mass	0% (n = 0)	2.0% (n = 6)	0.207
Intestinal cancer	0% (n = 0)	1.0% (n = 3)	0.374
Cesarean section	0% (n = 0)	0.7% (n = 2)	0.469
Ectopic pregnancy	0% (n = 0)	0.3% (n = 1)	0.609
Paravertebral cyst	0% (n = 0)	0.3% (n = 1)	0.609
Spleenic mass	0% (n = 0)	0.3% (n = 1)	0.609

* Neoplasm also includes non-invasive entities such as cervical cancer-in situ, endometrial hyperplasia, and borderline ovarian tumors.

**Table 3:** Phenotype and visual pattern distribution of endolsapingiosis lesions in the own and systematic review population.

			Own Population	Systematic Review	
			(n = 50)	(n = 99)	
Size mean (mm)			3.6 (range 1–40)	48.5 (range 2–250)	*p* < 0.001 *
Shape					
	symmetric		64% (n = 32)	36.4% (n = 36)	*p* = 0.001
	irregular		36% (n = 18)	63.6% (n = 63)	
Color					
	transparent		48% (n = 24)	31.3% (n = 31)	*p* = 0.005
	white		22% (n = 11)	10.1% (n = 10)	
	yellow		10% (n = 5)	15.2% (n = 15)	
	light red		16% (n = 8)	18.2% (n = 18)	
	dark red/brown		4% (n = 2)	25.3% (n = 25)	
Height					
	flat		70% (n = 35)	8.1% (n = 8)	*p* < 0.001
	polypous		22% (n = 11)	15.2% (n = 15)	
	cystic		8% (n = 4)	76.8% (n = 76)	
Surface					
	smooth		84% (n = 42)	88.9% (n = 88)	*p* = 0.398
	irregular		16% (n = 8)	11.1% (n = 11)	
Consistency					
	soft		88% (n = 44)	67.7% (n = 67)	*p* = 0.007
	solid		12% (n = 6)	32.3% (n = 32)	
Calcification					
	no		76% (n = 38)	87.9% (n = 78)	*p* = 0.063
	yes		24% (n = 12)	12.1% (n = 12)	
Adhesions					
	no		68% (n = 34/50)	91.9% (n = 91/99)	*p* = 0.002
	string		8% (n = 4/50)	1% (n = 1/99)	
	area		14% (n = 7/50)	3% (n = 3/99)	
	dense		10% (n = 5/50)	4% (n = 4/99)	
Endometriosis					
	no		74% (n = 37/50)	88.9% (n = 88/99)	*p* = 0.054
	peritoneal		16% (n = 8/50)	8.1% (n = 8/99)	
	deep		10% (n = 5/50)	3% (n = 3/99)	
Pattern type					
	1	vesicular	62% (n = 31/50)	8.1% (n = 8/99)	*p* < 0.001
	2	polypous	6% (n = 3/50)	11.1% (n = 11/99)	
	3	fimbrial like	12% (n = 6/50)	1% (n = 1/99)	
	4	cystic	10% (n = 5/50)	49.5% (n = 49/99)	
	5	unusual	10% (n = 5/50)	30.3% (n = 30/99)	

* Mann–Whitney-U test. All others were the Chi-square test.

**Table 4:** Anatomical distribution of endolsapingiosis lesions in the own and systematic review population.

		Own Population	Systematic Review	
		n = 77	n = 1174	*p*-Values
Urinary tract		19.5% (n = 15)	4.1% (n = 48)	<0.001
Bladder		19.5% (n = 15)	4% (n = 47)	<0.001
	Peritoneal	19.5% (n = 15)	1% (n = 12)	<0.001
	Deep	0% (n = 0)	3% (n = 35)	0.124
Ureter		0% (n = 0)	0.1% (n = 1)	0.798
Vagina		0% (n = 0)	0.1% (n = 1)	0.798
Uterus		6.5% (n = 5)	6.3% (n = 74)	0.947
Surface		6.5% (n = 5)	4.7% (n = 56)	0.496
Deep		0% (n = 0)	1.4% (n = 17)	0.288
Ovary		15.6% (n = 12)	23.2% (n = 272)	<0.001
Left		5.2% (n = 4)	2.6% (n = 30)	0.168
Right		10.4% (n = 8)	2.9%(n = 34)	<0.001
NOS		0% (n = 0)	17.7% (n = 208)	<0.001
Fallopian tube		15.6% (n = 12)	20.4% (n = 239)	<0.001
Left		6.5% (n = 5)	0.6% (n = 7)	<0.001
Right		9.1% (n = 7)	0.5% (n = 6)	<0.001
NOS		0% (n = 0)	19.3% (n = 226)	<0.001
Parametrium		11.7% (n = 9)	3.2% (n = 38)	<0.001
Left		7.8% (n = 6)	0.3% (n = 4)	<0.001
Right		3.9% (n = 3)	0.4% (n = 5)	<0.001
NOS		0% (n = 0)	2.5% (n = 29)	0.163
Rectovaginal septum		2.6% (n = 2)	0.1% (n = 1)	<0.001
Sacrouterine ligament		24.7% (n = 19)	1.2% (n = 14)	<0.001
Left		7.8% (n = 6)	0.4% (n = 5)	<0.001
Right		16.9% (n = 13)	0.5% (n = 6)	<0.001
NOS		0% (n = 0)	0.3% (n = 3)	0.657
Pelvic sidewall		18.2% (n = 14)	1.1% (n = 13)	<0.001
Left		9.1% (n = 7)	0.4% (n = 5)	<0.001
Right		6.5% (n = 5)	0.3% (n = 4)	<0.001
NOS		0% (n = 0)	0.3% (n = 4)	0.608
Douglas		20.7% (n = 16)	6.7% (n = 79)	<0.001
Peritoneal NOS		0% (n = 0)	7.3% (n = 86)	0.014
Retroperitoneum		0% (n = 0)	0.5% (n = 6)	0.529
Lumbar nerve root		0% (n = 0)	0.1% (n = 1)	0.798
Paravertebral		0% (n = 0)	0.2% (n = 2)	0.717
NOS		0% (n = 0)	0.3% (n = 3)	0.657
Abdominal wall		3.9% (n = 3)	1.2% (n = 14)	0.047
Left		2.6% (n = 2)	0.1% (n = 1)	<0.001
Right		1.3% (n = 1)	0% (n = 0)	<0.001
Umbilicus		0% (n = 0)	0.4% (n = 5)	0.566
Inguinal		0% (n = 0)	0.6% (n = 7)	0.497
NOS		0% (n = 0)	0.1% (n = 1)	0.798
Abdominal organs		10.4% (n = 8)	8% (n = 94)	0.459
Small bowel		0% (n = 0)	0.2% (n = 2)	0.717
Sigma/Colon		2.6% (n = 2)	1.2% (n = 15)	0.333
Appendix		1.3% (n = 1)	2.4% (n = 28)	0.539
Omentum		6.5% (n = 5)	5.5% (n = 65)	0.723
Spleen		0% (n = 0)	0.2% (n = 2)	0.717
Gallbladder/ductus choledochus		0% (n = 0)	0.2% (n = 2)	0.717
Pleura		0% (n = 0)	0.1% (n = 1)	0.798
Lymph node		7.8% (n = 6)	18.5% (n = 217)	<0.001
Inguinal		0% (n = 0)	0.3% (n = 3)	0.657
Pelvic		5.2% (n = 4)	3.9% (n = 46)	<0.001
Paraaortic		2.6% (n = 2)	0.8% (n = 10)	<0.001
Pelvi-abdominal NOS		0% (n = 0)	9.7% (n = 114)	0.004
Axillary		0% (n = 0)	3.3% (n = 39)	0.104
Neck		0% (n = 0)	0.3% (n = 4)	0.608
Mediastinal		0% (n = 0)	0.1% (n = 1)	0.798
Abdominal washing		0% (n = 0)	4.5% (n = 53)	0.057

NOS = not otherwise specified.

## Data Availability

The data presented in this study are available on request from the corresponding author. The data are not publicly available according to the requirements of the ethics committee.

## References

[1] Prentice L., Stewart A., Mohiuddin S., Johnson N.P. (2012). What is endosalpingiosis?. Fertil. Steril..

[2] Hesseling M.H., De Wilde R.L. (2000). Endosalpingiosis in laparoscopy. J. Am. Assoc. Gynecol. Laparosc..

[3] Sampson J.A. (1930). Postsalpingectomy endometriosis (endosalpingiosis). Am. J. Obs. Gynecol..

[4] Batt R.E., Yeh J. (2013). Müllerianosis: Four developmental (embryonic) mullerian diseases. Reprod Sci..

[5] Hermens M., van Altena A.M., Bulten J., Siebers A.G., Bekkers R.L.M. (2020). Increased association of ovarian cancer in women with histological proven endosalpingiosis. Cancer Epidemiol..

[6] Burney R.O., Giudice L.C. (2012). Pathogenesis and pathophysiology of endometriosis. Fertil. Steril..

[7] Esselen K.M., Terry K.L., Samuel A., Elias K.M., Davis M., Welch W.R., Muto M.G., Ng S.W., Berkowitz R.S. (2016). Endosalpingiosis: More than just an incidental finding at the time of gynecologic surgery?. Gynecol. Oncol..

[8] Chui M.H., Shih I.M. (2020). Oncogenic BRAF and KRAS mutations in endosalpingiosis. J. Pathol..

[9] Redwine D.B. (1987). Age-related evolution in color appearance of endometriosis. Fertil. Steril..

[10] von Elm E., Altman D.G., Egger M., Pocock S.J., Gøtzsche P.C., Vandenbroucke J.P., STROBE Initiative (2008). The Strengthening the Reporting of Observational Studies in Epidemiology (STROBE) statement: Guidelines for reporting observational studies. J. Clin. Epidemiol..

[11] Page M.J., McKenzie J.E., Bossuyt P.M., Boutron I., Hoffmann T.C., Mulrow C.D., Shamseer L., Tetzlaff J.M., Akl E.A., Brennan S.E. (2021). The PRISMA 2020 statement: An updated guideline for reporting systematic reviews. BMJ.

[12] Burla L., Kalaitzopoulos D.R., Mrozek A., Eberhard M., Samartzis N. (2022). How and where to expect endosalpingiosis intraoperatively. Fertil. Steril..

[13] Wells G.A., Shea B., O’Conell D., Peterson J., Welch V., Losos M., Tugwel P. (2000). The Newcastle Ottawa Scale (NOS) for Assessing the Quality of Nonrandomised Studies in Meta-Analyses.

[14] Moola S., Munn Z., Tufanaru C., Aromataris E., Munn Z. (2017). Systematic reviews of etiology and risk. Joanna Briggs Institute Rewiewer’s Manual for Evidence Synthesis.

[15] Doré N., Landry M., Cadotte M., Schürch W. (1980). Cutaneous Endosalpingiosis. Arch. Dermatol..

[16] Bryce R.L., Barbatis C., Charnock M. (1982). Endosalpingiosis in pregnancy. Case report. Br. J. Obstet. Gynaecol..

[17] Dallenbach-Hellweg G. (1987). Atypical endosalpingiosis: A case report with consideration of the differential diagnosis of glandular subperitoneal inclusions. Pathol. Res. Pract..

[18] Bazot M., Vacher Lavenu M.C., Bigot J.M. (1999). Imaging of endosalpingiosis. Clin. Radiol..

[19] Arai Y., Tsuzuki M., Okubo Y., Aizawa T., Miki M. (1999). A case of submucosal endosalpingiosis in the urinary bladder. Nihon Hinyokika Gakkai Zasshi..

[20] Santeusanio G., Ventura L., Partenzi A., Spagnoli L.G., Kraus F.T. (1999). Omental endosalpingiosis with endometrial-type stroma in a woman with extensive hemorrhagic pelvic endometriosis. Am. J. Clin. Pathol..

[21] Rondez R., Kunz J. (2000). Serous cystadenofibroma of the epiploic appendix. A tumor of the secondary müllerian system: Case report and review of the literature. Pathologe.

[22] Heatley M.K., Russell P. (2001). Florid cystic endosalpingiosis of the uterus. J. Clin. Pathol..

[23] Redondo P., Idoate M., Corella C. (2001). Cutaneous umbilical endosalpingiosis with severe abdominal pain. J. Eur. Acad. Dermatol. Venereol..

[24] McCluggage W.G., O’Rourke D., McElhenney C., Crooks M. (2002). Mullerian papilloma-like proliferation arising in cystic pelvic endosalpingiosis. Hum. Pathol..

[25] Edmondson J.D., Vogeley K.J., Howell J.D., Koontz W.W., Koo H.P., Amaker B. (2002). Endosalpingiosis of bladder. J. Urol..

[26] Heinig J., Gottschalk I., Cirkel U., Diallo R. (2002). Endosalpingiosis-an underestimated cause of chronic pelvic pain or an accidental finding? A retrospective study of 16 cases. Eur. J. Obstet. Gynecol. Reprod. Biol..

[27] Gerber T., Bontikous S., Smolka G., Vestring T., Schmidt D., Gickler W. (2002). Cystic lymphangioma with endosalpingiosis as a rare cause of gastrointestinal bleeding. Z. Gastroenterol..

[28] Chang Y., Tsai E.M., Yang C.H., Kuo C.H., Lee J.N. (2003). Multilobular cyst as endosalpingiosis of uterine serosa: A case report. Kaohsiung J. Med. Sci..

[29] Fukunaga M. (2004). Tumor-like cystic endosalpingiosis of the uterus with florid epithelial proliferation. A case report. APMIS.

[30] Perera G.K., Watson K.M., Salisbury J., Du Vivier A.W. (2004). Two cases of cutaneous umbilical endosalpingiosis. Br. J. Dermatol..

[31] Smith C., Sabet L., Izawa J.I. (2004). Management of endosalpingiosis of urinary bladder. Urology.

[32] Kajo K., Zúbor P., Macháleková K., Plank L., Visnovskỳ J. (2005). Tumor-like manifestation of endosalpingiosis in uterus: A case report. Pathol. Res. Pract..

[33] Lee S.N., Cho M.S., Kim S.C., Han W.S. (2005). Tumor-like multilocular cystic endosalpingiosis of the uterine serosa: Possible clinical and radiologic misinterpreted. Acta Obstet. Gynecol. Scand..

[34] Cunnick G.H., Pietzrak P., Richardson N.G., Ratcliffe N., Donaldson D.R. (2005). Multiple, large, benign peritoneal cysts—A case report. Int. J. Clin. Pract. Suppl..

[35] McCoubrey A., Houghton O., McCallion K., McCluggage W.G. (2005). Serous adenocarcinoma of the sigmoid mesentery arising in cystic endosalpingiosis. J. Clin. Pathol..

[36] Youssef A.H., Ganesan R., Rollason T.P. (2006). Florid cystic endosalpingiosis of the uterus. Histopathology.

[37] Koren J., Mensikova J., Mukensnabl P., Zamecnik M. (2006). Mullerianosis of the urinary bladder: Report of a case with suggested metaplastic origin. Virchows Arch..

[38] Tanahashi J., Kashima K., Daa T., Kondo Y., Kitano S., Yokoyama S. (2006). Florid cystic endosalpingiosis of the spleen. APMIS.

[39] Li W.M., Yang S.F., Lin H.C., Juan H.C., Wu W.J., Huang C.H., Wang C.J., Li C.C. (2007). Müllerianosis of ureter: A rare cause of hydronephrosis. Urology..

[40] Liang J.J., Malpica A., Broaddus R.R. (2007). Florid cystic endosalpingiosis presenting as an obstructive colon mass mimicking malignancy: Case report and literature review. J. Gastrointest. Cancer.

[41] Fukunaga M., Mistuda A., Shibuya K., Honda Y., Shimada N., Koike J., Sugimoto M. (2007). Retroperitoneal lymphangioleiomyomatosis associated with endosalpingiosis. APMIS.

[42] Cil A.P., Atasoy P., Kara S.A. (2008). Myometrial involvement of tumor-like cystic endosalpingiosis: A rare entity. Ultrasound Obs. Gynecol..

[43] Driss M., Zhioua F., Doghri R., Mrad K., Dhouib R., Romdhane K.B. (2009). Cotyledonoid dissecting leiomyoma of the uterus associated with endosalpingiosis. Arch Gynecol Obstet..

[44] Suarez-Vilela D., Izquierdo-Garcia F.M., Mendez-Alvarez J.R., Dominguez-Iglesias F. (2009). Florid cystic endosalpingiosis inside a uterine subserous leiomyoma. Pathology.

[45] Papavramidis T.S., Sapalidis K., Michalopoulos N., Karayannopoulou G., Cheva A., Papavramidis S.T. (2010). Umbilical endosalpingiosis: A case report. J. Med. Case Rep..

[46] Taneja S., Sidhu R., Khurana A., Sekhon R., Mehta A., Jena A. (2010). MRI appearance of florid cystic endosalpingiosis of the uterus: A case report. Korean J. Radiol..

[47] Batt R.E., Mhawech-Fauceglia P., Odunsi K., Yeh J. (2010). Pathogenesis of mediastinal paravertebral müllerian cysts of Hattori: Developmental endosalpingiosis-müllerianosis. Int. J. Gynecol. Pathol..

[48] Maniar K.P., Kalir T.L., Palese M.A., Unger P.D. (2010). Endosalpingiosis of the urinary bladder: A case of probable implantative origin with characterization of benign Fallopian tube immunohistochemistry. Int. J. Surg. Pathol..

[49] Olivia Vella J.E., Nair N., Ferryman S.R., Athavale R., Latthe P., Hirschowitz L. (2011). Müllerianosis of the urinary bladder. Int. J. Surg. Pathol..

[50] Patonay B., Semer D., Hong H. (2011). Florid cystic endosalpingiosis with extensive peritoneal involvement and concurrent bilateral ovarian serous cystadenoma. J. Obstet. Gynaecol..

[51] Rosenberg P., Nappi L., Santoro A., Bufo P., Greco P. (2011). Pelvic mass-like florid cystic endosalpingiosis of the uterus: A case report and a review of literature. Arch. Gynecol. Obstet..

[52] Zapardiel I., Tobias-Gonzalez P., de Santiago J. (2012). Endosalpingiosis mimicking recurrent ovarian carcinoma. Taiwan J. Obs. Gynecol..

[53] Kudva R., Hegde P. (2012). Mullerianosis of the urinary bladder. Indian J. Urol..

[54] Bermejo R., Gómez A., Galiana N., Campos A., Puente R., Bas E., Díaz-Caneja C. (2012). Peritoneal mullerian tumor-like (endosalpingiosis-leiomyomatosis peritoneal): A hardly known entity. Case Rep. Obstet. Gynecol..

[55] Scheel A.H., Frasunek J., Meyer W., Ströbel P. (2013). Cystic endosalpingiosis presenting as chronic back pain, a case report. Diagn. Pathol..

[56] Oida T., Otoshi T., Kobayashi K., Madono K., Momohara C., Imamura R., Takada S., Matsumiya K., Oka K., Tsujimoto M. (2013). Endocervicosis/endosalpingiosis of the bladder: A case report. Hinyokika Kiyo. Acta Urol. Jpn..

[57] Mishima T., Harada J., Kawa G., Okada T. (2013). [A case of endosalpingiosis in submucosa of the urinary bladder]. Hinyokika Kiyo..

[58] Maeda K., Kojima F., Ishida M., Iwai M., Kagotani A., Kawauchi A. (2014). Müllerianosis and endosalpingiosis of the urinary bladder: Report of two cases with review of the literature. Int. J. Clin. Exp. Pathol..

[59] Yığıt S., Dere Y., Yetımalar H., Etıt D. (2014). Tumor-like cystic endosalpingiosis in the myometrium: A case report and a review of the literature. Turk. J. Pathol..

[60] Singhania N., Janakiraman N., Coslett D., Ahmad N. (2014). Endosalpingiosis in conjunction with ovarian serous cystadenoma mimicking metastatic ovarian malignancy. Am. J. Case Rep..

[61] Goodman S., Khan A. (2014). Florid Cystic Endosalpingiosis. Int. J. Surg. Pathol..

[62] Cheung K.W., Cheung V.Y. (2015). Coexisting endosalpingiosis and subserous adenomyosis. J. Minim. Invasive Gynecol..

[63] Partyka L., Steinhoff M., Lourenco A.P. (2014). Endosalpingiosis presenting as multiple pelvic masses. J. Obstet. Gynaecol..

[64] Hemalatha A.L., Ashok K.P., Anoosha K., Indira C.S. (2014). Cystic endosalpingiosis of uterine parametrium- a scarcely encountered and sparsely documented entity. J. Clin. Diagn. Res..

[65] Lui M.W., Ngu S.F., Cheung V.Y. (2014). Mullerian cyst of the uterus misdiagnosed as ovarian cyst on pelvic sonography. J. Clin. Ultrasound..

[66] Singh N., Murali S., Zangmo R. (2014). Florid cystic endosalpingiosis, masquerading as malignancy in a young patient: A brief review. BMJ Case Rep..

[67] Kaneda S., Fujii S., Nosaka K., Inoue C., Tanabe Y., Matsuki T., Ogawa T. (2015). MR imaging findings of mass-forming endosalpingiosis in both ovaries: A case report. Abdom Imaging.

[68] Morales-Roselló J., Pamplona-Bueno L., Montero-Balaguer B., Desantes-Real D., Perales-Marín A. (2016). Florid Cystic Endosalpingiosis (Müllerianosis) in Pregnancy. Case Rep. Obstet. Gynecol..

[69] Satgunaseelan L., Russell P., Phan-Thien K.C., Tran K., Sinclair E. (2016). Perineural space infiltration by endosalpingiosis. Pathology.

[70] Stanimir M., ChiuŢu L.C., Wese S., Milulescu A., Nemeş R.N., Bratu O.G. (2016). Müllerianosis of the urinary bladder: A rare case report and review of the literature. Rom. J. Morphol. Embryol..

[71] Zangmo R., Singh N., Kumar S., Vatsa R. (2017). Second Look of Endosalpingiosis: A Rare Entity. J. Obstet. Gynaecol. India.

[72] Mulayim B., Serin N., Karatas S., Celik B. (2017). Cystic Endosalpingiosis of Uterus and Ovary Found on Laparoscopy: Disease of Haze. J. Minim. Invasive Gynecol..

[73] Nguyen B.D., McCullough A.E. (2017). Gastrointestinal: Cystic endosalpingiosis of the spleen: CT, MR, and US imaging. J. Gastroenterol. Hepatol..

[74] Im S., Park H.S., Cho U., Yoo C., Jung J.-H., Yoo J., Choi H.J. (2017). Florid cystic endosalpingiosis associated with a retroperitoneal leiomyoma mimicking malignancy: A case report. Int. J. Clin. Exp. Pathol..

[75] Quirante F.P., Montorfano L.M., Serrot F., E Billington M., Da Silva G., Menzo E.L., Szomstein S., Rosenthal R.J. (2017). The case of the missing appendix: A case report of appendiceal intussusception at the site of colonic mullerianosis. Gastroenterol. Rep. (Oxf)..

[76] Câmara S., Mendinhos G., Madureira R., Martins A., Veríssimo C. (2017). Vaginal Endosalpingiosis Case Report: A Rare Entity Presenting as Intermenstrual Bleeding. Case Rep. Obstet. Gynecol..

[77] Hattori Y., Sentani K., Matsuoka N., Nakayama H., Hattori T., Kudo Y., Yasui W. (2018). Intramural florid cystic endosalpingiosis of the uterus after menopause. Pol. J. Pathol..

[78] Nixon K.E., Kenneth Schoolmeester J., Bakkum-Gamez J.N. (2018). Florid cystic endosalpingiosis with uterine preservation and successful assisted reproductive therapy. Gynecol. Oncol. Rep..

[79] Gilbert N., Guo X., Bauer J., Hennig M., Kümpers C., Merseburger A.S. (2018). Intravesical salpingiosis: Case report and review of the literature. Aktuelle Urol..

[80] Wang C.J., Li Y.C., Jung S.M., Liao Y.H., Huang Y.T. (2019). Masslike Cystic Endosalpingiosis in the Uterine Myometrium. J. Minim. Invasive Gynecol..

[81] Yang M., Li Y., Chen M., Chen J., Kung F.T. (2019). Uterine endosalpingiosis: Case report and review of the literature. Taiwan J. Obs. Gynecol..

[82] Saha A., Saha K., Mukhopadhyay J. (2019). Intramyometrial cystic endosalpingiosis-a rare entity in gynecological pathology: A case report and brief review of the literature. Indian J. Pathol. Microbiol..

[83] Niwa K., Sakamoto K., Goto M., Kojima Y., Takahashi M., Ishiyama S., Kawai M., Okazawa Y., Tomita N., Seki E. (2020). A case of endosalpingiosis in the lymph nodes of the mesocolon. Surg. Case Rep..

[84] Hortu İ., Arı S.A., Özçeltik G., Şahin C., Ergenoğlu A.M., Akercan F. (2020). Laparoscopic view of endosalpingiosis in a woman with dermoid cyst and endometriosis. J. Turk. Ger. Gynecol. Assoc..

[85] Mahdavi F.S., Tavallaei M., Ketabforoush A.H.M.E., Bahadorinia M. (2020). Paratubal endosalpingiosis: A case report. Int. J. Surg. Case Rep..

[86] Maheshwari S., Bhat V., Gadabanahalli K., Raju N., Kulkarni P. (2020). Endosalpingiosis of urinary bladder: Report on a rare entity. BJR Case Rep..

[87] Fernandez H., Dupeux M., Paris M., Sauvan M. (2021). Florid Cystic Endosalpingiosis and Adenomyosis of the Uterus Mimicking Malignancy. J. Minim. Invasive Gynecol..

[88] Talia K.L., Fiorentino L., Scurry J., McCluggage W.G. (2020). A Clinicopathologic Study and Descriptive Analysis of “Atypical Endosalpingiosis”. Int. J. Gynecol Pathol..

[89] Peixinho C., Machado-Neves R., Silva P.T., Bernardes J., Silva A.C., Amaro T. (2021). Hysteroscopic Findings Related with the Assessment and Treatment of Uterine Florid Cystic Endosalpingiosis: A Case Report and Review of All the Published Cases. Acta Med. Port..

[90] Subbaiah M., Toi P.C., Dorairajan G., Stephen S.N. (2020). Cystic Uterine Endosalpingiosis in a Patient with Carcinoma Endometrium. J. Midlife Health.

[91] Mahajan A.D., Mahajan S.A., Kulkarni D. (2020). Coexistence of malacoplakia and mullerianosis in the urinary bladder: An uncommon pathology. Indian J. Urol..

[92] Fakhralddin S.S., Mahmood S.N., Qader D.K., Ali A.A., Kakamad F.H., Salih A.M., Abdullah H.O. (2021). Mullerianosis of the urinary bladder; A case report. Int. J. Surg. Case Rep..

[93] Bocchialini T., Ziglioli F., Palmieri G., Barbieri A., Infranco A., Milandri R., Simonetti E., Ferretti S., Maestroni U. (2021). Müllerianosis of the urinary bladder may simulate a bladder cancer: A case report. Acta Biomed..

[94] Keihanian T., Kumar S.R., Ronquillo N., Amin S. (2021). A rare case of endosalpingiosis masquerading as a pedunculated subepithelial colonic nodule: Utility of EUS and endoscopic resection. Gastrointest. Endosc..

[95] D’Ovidio V., Maggi D., Bruno G., Fratoni S., Guazzaroni M. (2021). An Extragenital Colonic Salpingiosis. J. Gastrointestin. Liver Dis..

[96] Muirhead F.C., Lee H.L., Singh R. (2021). Ovarian endosalpingiosis mimicking hydrosalpinges. Unexpected intraoperative findings and a diagnostic rollercoaster. J. Surg. Case Rep..

[97] Busuttil G., German K., DeGaetano J., Scerri A.P. (2012). The menstruating bladder, an unusual cause of haematuria. Malta Med. J..

[98] Sanchez-Merino J.-M., Guillan-Maquieira C., Alvarez Garcia A., Mendez-Diaz C., Sanchez Rodriguez-Losada J., Chantada Abal V. (2014). Mullerianosis vesical. Prog. Obstet. Y Ginecol..

[99] Del Carmen S., Rodriguez M., Gomez M.A., Cruz M.A., Nunez M.A., Sacho M. (2015). Müllerianosis with Intestinal Metaplasia: A Case Report. Turk. J. Pathol..

[100] Manucha V., Azar A., Shwayder J.M., Hudgens J.L., Lewin J. (2015). Cystic adenomatoid tumor of the uterus. J. Cancer Res. Ther..

[101] Agrawal A., Darakh P., Patil M., Mahajan A. (2018). Mullerianosis of Urinary Bladder: A Great Impersonator of Malignant Urinary Bladder Tumours. Int. J. Contemp. Med. Res..

[102] Balthazar P., Kearns C., Zapparoli M. (2021). Endosalpingiosis. Radiographics.

[103] Almatrafi M.H., Alhazmi A.M., Almosaieed B.N. (2020). Mullerianosis of the urinary bladder. Urol. Case Rep..

[104] Ernst A., Aguilera E., Dabancens A. (1981). [Oviductal physiological alterations and endosalpingiosis (author’s transl)]. Reproduccion.

[105] Zinsser K.R., Wheeler J.E. (1982). Endosalpingiosis in the omentum: A study of autopsy and surgical material. Am. J. Surg. Pathol..

[106] Shen S.C., Bansal M., Purrazzella R., Malviya V., Strauss L. (1983). Benign glandular inclusions in lymph nodes, endosalpingiosis, and salpingitis isthmica nodosa in a young girl with clear cell adenocarcinoma of the cervix. Am. J. Surg. Pathol..

[107] Yeung H.H., Bannatyne P., Russell P. (1983). Adenocarcinoma of the fallopian tubes: A clinicopathological study of eight cases. Pathology.

[108] McCaughey W.T., Kirk M.E., Lester W., Dardick I. (1984). Peritoneal epithelial lesions associated with proliferative serous tumours of ovary. Histopathology.

[109] Carlson G.J., Samuelson J.J., Dehner L.P. (1986). Cytologic diagnosis of florid peritoneal endosalpingiosis. A case report. Acta Cytol..

[110] Michael H., Roth L.M. (1986). Invasive and noninvasive implants in ovarian serous tumors of low malignant potential. Cancer.

[111] Sidawy M.K., Silverberg S.G. (1987). Endosalpingiosis in female peritoneal washings: A diagnostic pitfall. Int. J. Gynecol. Pathol..

[112] Dienemann D., Pickartz H. (1987). So-called peritoneal implants of ovarian carcinomas. Problems in differential diagnosis. Pathol. Res. Pract..

[113] Cajigas A., Axiotis C.A. (1990). Endosalpingiosis of the vermiform appendix. Int. J. Gynecol. Pathol..

[114] Tohya T., Nakamura M., Fukumatsu Y., Katabuchi H., Matsuura K., Itoh M., Okamura H. (1991). Endosalpingosis in the pelvic peritoneum and pelvic lymph nodes. Nihon Sanka Fujinka Gakkai Zasshi..

[115] Biscotti C.V., Hart W.R. (1992). Peritoneal serous micropapillomatosis of low malignant potential (serous borderline tumors of the peritoneum). A clinicopathologic study of 17 cases. Am. J. Surg. Pathol..

[116] Padberg B.C., Stegner H.E., von Sengbusch S., Arps H., Schröder S. (1992). DNA-cytophotometry and immunocytochemistry in ovarian tumours of borderline malignancy and related peritoneal lesions. Virchows Arch A Pathol. Anat. Histopathol..

[117] Kadar N., Krumerman M. (1995). Possible metaplastic origin of lymph node “metastases” in serous ovarian tumor of low malignant potential (borderline serous tumor). Gynecol. Oncol..

[118] Horn L.C., Bilek K. (1995). Frequency and histogenesis of pelvic retroperitoneal lymph node inclusions of the female genital tract. An immunohistochemical study of 34 cases. Pathol. Res Pract..

[119] Khare V.K., Martin D.C. (1995). Anecdotal association of endosalpingiosis with chlamydia trachomatis IgG titers and Fitz-Hugh-Curtis adhesions. J. Am. Assoc. Gynecol. Laparosc..

[120] Grouls V. (1996). [Proliferating serous papillary cystadenoma of the borderline type in myometrium of the fundus uteri]. Geburtshilfe Frauenheilkd..

[121] Silva E.G., Tornos C., Zhuang Z., Merino M.J., Gershenson D.M. (1998). Tumor recurrence in stage I ovarian serous neoplasms of low malignant potential. Int. J. Gynecol. Pathol..

[122] Kupfer M.C., Ralls P.W., Fu Y.S. (1998). Transvaginal sonographic evaluation of multiple peripherally distributed echogenic foci of the ovary: Prevalence and histologic correlation. AJR Am. J. Roentgenol..

[123] Yang X.J., Lecksell K., Epstein J.I. (1999). Can immunohistochemistry enhance the detection of micrometastases in pelvic lymph nodes from patients with high-grade urothelial carcinoma of the bladder?. Am. J. Clin. Pathol..

[124] Tsuchiya A., Kikuchi Y., Matsuoka T., Yashima R., Abe R., Suzuki T. (1999). Endosalpingiosis of nonmetastatic lymph nodes along the stomach in a patient with early gastric cancer: Report of a case. Surg. Today.

[125] McCluggage W.G., Weir P.E. (2000). Paraovarian cystic endosalpingiosis in association with tamoxifen therapy. J. Clin. Pathol..

[126] Jiménez-Heffernan J.A., Sánchez-Piedra D., Bernaldo de Quiros L., Martínez V. (2000). Endosalpingiosis (müllerianosis) of the bladder: A potential source of error in urinary cytology. Cytopathology.

[127] Risberg B., Davidson B., Dong H.P., Nesland J.M., Berner A. (2000). Flow cytometric immunophenotyping of serous effusions and peritoneal washings: Comparison with immunocytochemistry and morphological findings. J. Clin. Pathol..

[128] Matsui K., Travis W.D., Gonzalez R., Terzian J.A., Rosai J., Moss J., Ferrans V.J. (2001). Association of lymphangioleiomyomatosis (LAM) with endosalpingiosis in the retroperitoneal lymph nodes: Report of two cases. Int. J. Surg. Pathol..

[129] McCluggage W.G., Clements W.D. (2001). Endosalpingiosis of the colon and appendix. Histopathology.

[130] Malpica A., Deavers M.T., Gershenson D., Tortolero-Luna G., Silva E.G. (2001). Serous tumors involving extra-abdominal/extra-pelvic sites after the diagnosis of an ovarian serous neoplasm of low malignant potential. Am. J. Surg. Pathol..

[131] Kalir T., Wang B.Y., Goldfischer M., Haber R.S., Reder I., Demopoulos R., Cohen C.J., Burstein D.E. (2002). Immunohistochemical staining of GLUT1 in benign, borderline, and malignant ovarian epithelia. Cancer.

[132] Camatte S., Morice P., Atallah D., Pautier P., Lhommé C., Haie-Meder C., Duvillard P., Castaigne D. (2002). Lymph node disorders and prognostic value of nodal involvement in patients treated for a borderline ovarian tumor: An analysis of a series of 42 lymphadenectomies. J. Am. Coll. Surg..

[133] Vilos G.A., Vilos A.W., Haebe J.J. (2002). Laparoscopic findings, management, histopathology, and outcome of 25 women with cyclic leg pain. J. Am. Assoc. Gynecol. Laparosc..

[134] Hunt J.L., Lynn A.A. (2002). Histologic features of surgically removed fallopian tubes. Arch Pathol Lab Med..

[135] Fausett M.B., Zahn C.M., Kendall B.S., Barth W.H. (2002). The significance of psammoma bodies that are found incidentally during endometrial biopsy. Am. J. Obstet. Gynecol..

[136] Shappell H.W., Riopel M.A., Smith Sehdev A.E., Ronnett B.M., Kurman R.J. (2002). Diagnostic criteria and behavior of ovarian seromucinous (endocervical-type mucinous and mixed cell-type) tumors: Atypical proliferative (borderline) tumors, intraepithelial, microinvasive, and invasive carcinomas. Am. J. Surg. Pathol..

[137] Diebold J., Seemüller F., Löhrs U. (2003). K-RAS mutations in ovarian and extraovarian lesions of serous tumors of borderline malignancy. Lab Invest..

[138] Yoo J., Choi H.J., Kang S.J. (2003). M llerian-Type Gland Inclusions in Pelvic Lymph Nodes Mimicking Metastasis: A Case Report and Review of the Literature. Cancer Res. Treat..

[139] Carrick K.S., Milvenan J.S., Albores-Saavedra J. (2003). Serous tumor of low malignant potential arising in inguinal endosalpingiosis: Report of a case. Int. J. Gynecol. Pathol..

[140] Sadeghi S., Ylagan L.R. (2004). Pelvic washing cytology in serous borderline tumors of the ovary using ThinPrep: Are there cytologic clues to detecting tumor cells?. Diagn Cytopathol..

[141] Brown D.L., Frates M.C., Muto M.G., Welch W.R. (2004). Small echogenic foci in the ovaries: Correlation with histologic findings. J. Ultrasound Med..

[142] Kobayashi T.K., Moritani S., Urabe M., Bamba M., Ueda M., Nishino T., Muramatsu M., Kaneko C. (2004). Cytologic diagnosis of endosalpingiosis with pregnant women presenting in peritoneal fluid: A case report. Diagn Cytopathol..

[143] Lee E.S., Leong A.S.-Y., Kim Y.-S., Lee J.-H., Kim I., Ahn G.H., Kim H.S., Chun Y.K. (2006). Calretinin, CD34, and alpha-smooth muscle actin in the identification of peritoneal invasive implants of serous borderline tumors of the ovary. Mod. Pathol..

[144] Nascu P.C., Vilos G.A., Ettler H.C., Abu-Rafea B., Hollet-Caines J., Ahmad R. (2006). Histopathologic findings on uterosacral ligaments in women with chronic pelvic pain and visually normal pelvis at laparoscopy. J. Minim. Invasive Gynecol..

[145] McKenney J.K., Balzer B.L., Longacre T.A. (2006). Lymph node involvement in ovarian serous tumors of low malignant potential (borderline tumors): Pathology, prognosis, and proposed classification. Am. J. Surg. Pathol..

[146] Mesquita I., Encinas A., Gradil C., Davide J., Daniel J., Graça L.M., Teixeira M. (2007). Endosalpingiosis of choledochal duct. Surgery.

[147] Pollheimer M.J., Leibl S., Pollheimer V.S., Ratschek M., Langner C. (2007). Cystic endosalpingiosis of the appendix. Virchows Arch..

[148] Tallón-Aguilar L., Olano-Acosta C., López-Porras M., Flores-Cortés M., Pareja-Ciuró F. (2009). Endosalpingiosis of the appendix. Cir. Esp..

[149] Hui J.P., Li A., Li H.L. (2009). Lymphangioleiomyomatosis with florid endosalpingiosis. AJR Am. J. Roentgenol..

[150] Hsu M., Young R.H., Misdraji J. (2009). Ruptured appendiceal diverticula mimicking low-grade appendiceal mucinous neoplasms. Am. J. Surg. Pathol..

[151] Lee K.M., Wong C., Russell P. (2009). Pelvic lymphangioleiomyomatosis associated with endosalpingiosis. Pathology.

[152] Herce J.M., Luque V.R., Luque J.A., García P.M., Gómez D.D. (2009). Association of retroperitoneal lymphangioleiomyomatosis with endosalpingiosis: A case report. Cases J..

[153] Corben A.D., Nehhozina T., Garg K., Vallejo C.E., Brogi E. (2010). Endosalpingiosis in axillary lymph nodes: A possible pitfall in the staging of patients with breast carcinoma. Am. J. Surg. Pathol..

[154] Djordjevic B., Clement-Kruzel S., Atkinson N.E., Malpica A. (2010). Nodal endosalpingiosis in ovarian serous tumors of low malignant potential with lymph node involvement: A case for a precursor lesion. Am. J. Surg. Pathol..

[155] Karafin M., Parwani A., Netto G.J., Illei P.B., Epstein J.I., Ladanyi M., Argani P. (2011). Diffuse expression of PAX2 and PAX8 in the cystic epithelium of mixed epithelial stromal tumor, angiomyolipoma with epithelial cysts, and primary renal synovial sarcoma: Evidence supporting renal tubular differentiation. Am. J. Surg. Pathol..

[156] Yang Q., Wang H., Cho H.Y., Jung S.J., Kim K.-R., Ro J.Y., Shen S.S. (2011). Carcinoma of müllerian origin presenting as colorectal cancer: A clinicopathologic study of 13 Cases. Ann. Diagn. Pathol..

[157] Li J., Abushahin N., Pang S., Xiang L., Chambers S.K., Fadare O., Kong B., Zheng W. (2011). Tubal origin of ‘ovarian’ low-grade serous carcinoma. Mod. Pathol..

[158] Kurman R.J., Vang R., Junge J., Hannibal C.G., Kjaer S.K., Shih I.M. (2011). Papillary tubal hyperplasia: The putative precursor of ovarian atypical proliferative (borderline) serous tumors, noninvasive implants, and endosalpingiosis. Am. J. Surg. Pathol..

[159] Sarode V.R., Euhus D., Thompson M., Peng Y. (2011). Atypical endosalpingiosis in axillary sentinel lymph node: A potential source of false-positive diagnosis of metastasis. Breast J..

[160] Malpica A., Sant’Ambrogio S., Deavers M.T., Silva E.G. (2012). Well-differentiated papillary mesothelioma of the female peritoneum: A clinicopathologic study of 26 cases. Am. J. Surg. Pathol..

[161] Sneige N., Dawlett M., Kologinczak T., Guo M. (2013). Endosalpingiosis in Peritoneal Washings in Women with Benign Gynecologic Conditions. Cancer Cytopathol..

[162] Landon G., Stewart J., Deavers M., Lu K., Sneige N. (2012). Peritoneal washing cytology in patients with BRCA1 or BRCA2 mutations undergoing risk-reducing salpingo-oophorectomies: A 10-year experience and reappraisal of its clinical utility. Gynecol. Oncol..

[163] Djordjevic B., Malpica A. (2012). Ovarian serous tumors of low malignant potential with nodal low-grade serous carcinoma. Am. J. Surg. Pathol..

[164] Suárez-Vilela D., Izquierdo F.M., Riera-Velasco J.R., Escobar-Stein J. (2012). Endosalpingiosis can mimic malignant glands and result in a false positive mesorectal resection margin. Virchows Arch..

[165] Pun J.J., Vilos G.A., Ettler H.C., Marks J., Vilos A.G., Abu-Rafea B. (2012). Granulosa cells in the uterosacral ligament: Case report and review of the literature. J. Minim. Invasive Gynecol..

[166] Stojanovic M., Brasanac D., Stojicic M. (2013). Cutaneous inguinal scar endosalpingiosis and endometriosis: Case report with review of literature. Am. J. Dermatopathol..

[167] García Sánchez C., Ocón Revuelta E.M., Fontillón M., Argüelles Salido E., Medina López R.A. (2013). [Endosalpingiosis of the bladder. A case report]. Arch. Esp. Urol..

[168] Mincik I., Mytnik M., Straka L., Breza J., Vilcha I. (2013). Inflammatory pseudotumour of urinary bladder—A rare cause of massive macroscopic haematuria. Bratisl. Lek. Listy..

[169] Salehi A.H., Omeroglu G., Kanber Y., Omeroglu A. (2013). Endosalpingiosis in axillary lymph nodes simulating metastatic breast carcinoma: A potential diagnostic pitfall. Int. J. Surg. Pathol..

[170] Wilsher M., Snook K.L. (2014). Endosalpingiosis in an axillary lymph node with synchronous micro-metastatic mammary carcinoma. Pathology.

[171] Carney E., Cimino-Mathews A., Argani C., Kronz J., Vang R., Argani P. (2014). A subset of nondescript axillary lymph node inclusions have the immunophenotype of endosalpingiosis. Am. J. Surg. Pathol..

[172] Park J., Kim T.H., Lee H.H., Chung S.H., Jeon D.S. (2014). Endosalpingiosis in postmenopausal elderly women. J. Menopausal. Med..

[173] Ozerdem U., Hoda S.A. (2015). Endosalpingiosis of axillary sentinel lymph node: A mimic of metastatic breast carcinoma. Breast J..

[174] Nomani L., Calhoun B.C., Biscotti C.V., Grobmyer S.R., Sturgis C.D. (2016). Endosalpingiosis of Axillary Lymph Nodes: A Rare Histopathologic Pitfall with Clinical Relevance for Breast Cancer Staging. Case Rep. Pathol..

[175] Chakhtoura G., Nassereddine H., Gharios J., Khaddage A. (2016). Isolated endosalpingiosis of the appendix in an adolescent girl. Gynecol. Obstet. Fertil..

[176] Russell P., van der Griend R., Anderson L., Yu B., O’Toole S., Simcock B. (2016). Evidence for lymphatic pathogenesis of endosalpingiosis. Pathology.

[177] Russell P., Anderson L. (2016). Evidence for lymphatic pathogenesis of endosalpingiosis: The more things change, the more they stay the same. Pathology.

[178] Groth J.V., Prabhu S., Wiley E. (2016). Coexistent Isolated Tumor Cell Clusters of Infiltrating Lobular Carcinoma and Benign Glandular Inclusions of Müllerian (Endosalpingiosis) Type in an Axillary Sentinel Node: Case Report and Review of the Literature. Appl. Immunohistochem. Mol. Morphol..

[179] Gallan A.J., Antic T. (2016). Benign müllerian glandular inclusions in men undergoing pelvic lymph node dissection. Hum. Pathol..

[180] Barresi V., Barns D., Grundmeyer Rd R., Rodriguez F.J. (2017). Cystic endosalpingiosis of lumbar nerve root: A unique presentation. Clin. Neuropathol..

[181] Karpathiou G., Da Cruz V., Patoir A., Forest F., Hag B., Tiffet O., Peoc’h M. (2017). Mediastinal cyst of müllerian origin: Evidence for developmental endosalpingiosis. Pathology.

[182] Pérez Sánchez L.E., Hernández Barroso M., Hernández Hernández G., Soto Sánchez A., Barrera Gómez M. (2017). Endosalpingiosis as an obstructive entity simulating a sigma neoplasm. Cir. Esp..

[183] Demir M.K., Savas Y., Furuncuoglu Y., Cevher T., Demiral S., Tabandeh B., Aslan M. (2017). Imaging Findings of the Unusual Presentations, Associations and Clinical Mimics of Acute Appendicitis. Eurasian J. Med..

[184] Iida Y., Tabata J., Yorozu T., Kitai S., Ueda K., Saito M., Yanaihara N., Yamada K., Okamoto A. (2017). Polypoid endometriosis of the ovary and müllerianosis of pelvic lymph nodes mimicking an ovarian carcinoma with lymph node metastasis. Int. Cancer Conf. J..

[185] Dall C.P., Sharp D.S., Zynger D.L. (2017). Benign Müllerian Inclusions in Lymphadenectomies for Renal Cell Carcinoma: A Radiologic and Pathologic Mimic of Metastases. Clin. Genitourin. Cancer.

[186] Amir R.A.R., Taheini K.M., Sheikh S.S. (2018). Mullerianosis of the Urinary Bladder: A Case Report. Case Rep. Oncol..

[187] Porter K.R., Singh C., Neychev V. (2019). Endosalpingiosis of the Gallbladder: A Unique Complication of Ruptured Ectopic Pregnancy. Cureus.

[188] Shiino S., Yoshida M., Jimbo K., Asaga S., Takayama S., Maeshima A., Tsuda H., Kinoshita T., Hiraoka N. (2019). Two rare cases of endosalpingiosis in the axillary sentinel lymph nodes: Evaluation of immunohistochemical staining and one-step nucleic acid amplification (OSNA) assay in patients with breast cancer. Virchows Arch..

[189] Noor M., Chen A., Gonzalez R.S. (2019). Clinicopathologic findings in gynecologic proliferations of the appendix. Hum. Pathol..

[190] Cox H.Y., Alhatem A., Barlog L., Heller D. (2020). A Rare Mimic of Malignancy: Papillary Endosalpingiosis. Int. J. Surg. Pathol..

[191] Sah S., Fulmali R., McCluggage W.G. (2020). Low-grade Serous Carcinoma Arising in Inguinal Nodal Endosalpingiosis: Report of 2 Cases and Literature Review. Int. J. Gynecol. Pathol..

[192] Moreno G., Jorns J.M. (2020). Endosalpingiosis and other benign epithelial inclusions in breast sentinel lymph nodes. Breast J..

[193] Silva E.G., Kim G., Bakkar R., Bozdag Z., Shaye-Brown A., Loghavi S., Stolnicu S., Hadareanu V., Bulgaru D., Cayax L.I. (2020). Histology of the normal ovary in premenopausal patients. Ann. Diagn. Pathol..

[194] Sancheti S., Somal P.K., Chaudhary D., Khandelwal S. (2020). Mullerianosis of urinary bladder: The great impersonator. Indian J. Pathol. Microbiol..

[195] Singh K., Sardana R., Quddus M.R., Harigopal M. (2021). Epithelium Involving Bilateral Axillary Lymph Nodes: Metastasis, Misplaced, or Mullerian!. Int. J. Surg. Pathol..

[196] Matsumiya H., Todo Y., Yamazaki H., Yamada R., Minowa K., Tsuruta T., Kurosu H., Minobe S., Kato H., Suzuki H. (2020). Diagnostic criteria of sentinel lymph node micrometastasis or macrometastasis based on tissue rinse liquid-based cytology in gynecological cancer. Int. J. Clin. Oncol..

[197] Lee H.L., Farrell R., Kamath V., Ho-Shon I., Yap F. (2020). Concordant PET/CT and ICG positive lymph nodes in endometrial cancer: A case of mistaken identity. J. Surg. Case Rep..

[198] Sunde J., Wasickanin M., Katz T.A., Wickersham E.L., Steed D.O.E., Simper N. (2020). Prevalence of endosalpingiosis and other benign gynecologic lesions. PLoS ONE..

[199] Leivo M.Z., Tacha D.E., Hansel D.E. (2021). Expression of uroplakin II and GATA-3 in bladder cancer mimickers: Caveats in the use of a limited panel to determine cell of origin in bladder lesions. Hum. Pathol..

[200] Bonin M., Juncos A.C., Ganzer L.M., Cardona F.P., Abrego M. (2021). Uterine Florid Cystic Endosalpingiosis with Conservative Surgery. J. Minim. Invasive Gynecol..

[201] Tutschka B.G., Lauchlan S.C. (1980). Endosalpingiosis. Obstet. Gynecol..

[202] Mcintosh A., Teo U.L., Millan D.W.M., Alexander-Sefre F. (2008). Endosalpingiosis Masquerading as Ovarian Carcinoma and Presenting on Cervical Smear. Scott. Med. J..

[203] Segura Sanchez J., Solis Garcia E., Gonzalez Serrano T. (2008). Serous papillary adenocarcinoma of the sigmoid colon arising in cystic endosalpingiosis. Report of a case and review of the literature. Rev. Esp. Patol..

[204] Krentel H., Hucke J. (2010). Disseminated Hormone-Producing Leiomyomatosis after Laparoscopic Supracervical Hysterectomy: A Case Report. Geburtshilfe Frauenheikunde.

[205] Stolnicu S., Preda O., Kinga S., Marian C., Nicolau R., Andrei S., Nicolae A., Nogales F.F. (2011). Florid, papillary endosalpingiosis of the axillary lymph nodes. Breast J..

[206] Magill L., Rajan P., Zafar N., Seywright M., Hendry D. (2011). Endocervicosis and endosalpingiosis of the urinary bladder: A case report. Br. J. Med. Surg. Urol..

[207] Guan H., Rosenthal D.L., Erozan Y.S. (2012). Mullerianosis of the urinary bladder: Report of a case with diagnosis suggested in urine cytology and review of literature. Diagn Cytopathol..

[208] Im S., Jung J.H., Choi H.J., Kang C.S. (2015). Intramural florid cystic endosalpingiosis of the uterus: A case report and review of the literature. Taiwan J. Obstet. Gynecol..

[209] Bacalbasa N.B., Irina T.D. (2016). Lymph node involvement in endosalpingiosis. A case report and literature review. Ginecoeu.

[210] Pérez P.F., Fernández J.A., Sánchez B.C.G.I.P., Cañal P.L., Gil S.P., Gil S.P., Ferrera y.C.F. (2017). Dispositivos intratubáricos Essure^®^ asociados a dolor pélvico crónico y endosalpingiosis. Prog. De Obstet. Y Ginecol..

[211] Martín L.S., Trusso W.N., Puigoriol E.B., Weakner S.M., Cros N.E. (2018). Appendicular endosalpingiosis. Prog. Obstet. Y Ginecol..

[212] Ilnitsky S., Abu Rafea B., Vilos A.G., Vilos G.A. (2019). Pelvic Peritoneal Pockets: Distribution, Histopathology, and Clinical Significance. J. Obstet. Gynaecol. Can..

[213] Tudor J., Williams T.R., Myers D.T., Umar B. (2019). Appendiceal endosalpingiosis: Clinical presentation and imaging appearance of a rare condition of the appendix. Abdom. Radiol..

[214] Reyna-Villasmil E., Torres-Cepeda D., Rondon-Tapia M. (2020). Müllerianosis of cervix. Case report. Rev. Peru. Ginecol. Y Obstet..

[215] Sajnani J., Swan K. (2020). Endosalpingiosis: Clinical Presentation and Coexisting Pathology. Obstet. Gynecol..

[216] Fujii S., Inoue C., Mukuda N., Murakami A., Yamaji D., Yunaga H., Nosaka K. (2021). Magnetic resonance imaging findings of endosalpingiosis: A case report. Acta Radiol Open..

[217] White M.J., Vang R., Argani P., Cimino-Mathews A. (2021). Endosalpingiosis Is Negative for GATA3. Arch. Pathol. Lab. Med..

[218] O’Connor D., Byrnes K.G., Walsh K., O’Sullivan G., McHale T. (2021). Renal endometriosis mimicking a malignancy–a rare case of Reno-Mullerian fusion. Researchsquare.

[219] Hwang J.H., Song S.H., Shin B.K., Lee J.K., Lee N.W., Lee K.W. (2011). Primary clear cell carcinoma of a paratubal cyst: A case report with literature review. Aust. N Z J. Obstet. Gynaecol..

[220] Santoro A., Angelico G., Inzani F., Spadola S., Arciuolo D., Valente M., Fiorentino V., Mulè A., Scambia G., Zannoni G.F. (2020). The Many Faces of Endometriosis-Related Neoplasms in the Same Patient: A Brief Report. Gynecol. Obstet. Invest..

[221] Kurt S., Kandemir S., Yavuz O., Koyuncuoglu M., Ulukus E.C., Celiloglu M. (2020). Persistent tubal epithelium in ovaries after salpingectomy. Eur. J. Gynaecol. Oncol..

[222] Ong N.C.S., Maher P.J., Pyman J.M., Readman E., Gordon S. (2004). Endosalpingiosis, an unrecognized condition: Report and literature review. Gynecol. Surg..

[223] Ferenczy A., Talens M., Zoghby M., Hussain S.S. (1977). Ultrastructural studies on the morphogenesis of psammoma bodies in ovarian serous neoplasia. Cancer.

[224] Sun T., Pitman M.B., Torous V.F. (2021). Determining the significance of psammoma bodies in pelvic washings: A 10-year retrospective review. Cancer Cytopathol..

[225] Cheung K.W., Cheung V.Y. (2016). Response: Cystic Endosalpingiosis or Multicystic Mesothelioma?. J. Minim. Invasive Gynecol..

[226] Sun M., Zhao L., Weng Lao I., Yu L., Wang J. (2019). Well-differentiated papillary mesothelioma: A 17-year single institution experience with a series of 75 cases. Ann. Diagn. Pathol..

[227] Esselen K.M., Ng S.K., Hua Y., White M., Jimenez C.A., Welch W.R., Drapkin R., Berkowitz R.S., Ng S.W. (2014). Endosalpingiosis as it relates to tubal, ovarian and serous neoplastic tissues: An immunohistochemical study of tubal and Müllerian antigens. Gynecol. Oncol..

[228] Silva E.G., Lawson B.C., Ramalingam P., Liu J., Shehabeldin A., Marques-Piubelli M.L., Malpica A. (2022). Precursors in the ovarian stroma: Another pathway to explain the origin of ovarian serous neoplasms. Hum. Pathol..

[229] Redwine D.B. (2003). ‘Invisible’ microscopic endometriosis: A review. Gynecol. Obstet. Invest..

